# The Effect of Long-Term Aging on the Microstructure and Properties of a Novel Nickel-Based Powder Superalloy FGH4113A

**DOI:** 10.3390/ma17174175

**Published:** 2024-08-23

**Authors:** Jiangying Xiong, Chao Yin, Chong Wang, Ganjiang Feng, Jianzheng Guo

**Affiliations:** 1 State Key Laboratory of Powder Metallurgy, Central South University, Changsha 410083, China; xiong1988@csu.edu.cn (J.X.);; 2 Wedge Central South Research Institute Co., Ltd., Shenzhen 518045, China

**Keywords:** superalloy, long-term aging, metallographic, LCF

## Abstract

This study investigates the microstructural evolution and its effect on the fatigue performance of a novel nickel-based powder superalloy FGH4113A (WZ-A3) after long-term aging at 760 °C and 815 °C. The results show that long-term aging both at 760 °C and 815 °C has no significant effect on the grain size and morphology of the alloy. After aging at 760 °C for up to 2020 h, the size of the γ′ phase remains unchanged, and its morphology transitions from nearly square to nearly spherical. During long-term aging at 815 °C for 440 h, γ′ phase coarsening and spheroidizing occur simultaneously. With prolonged aging time, the size and spheroidization degree of the γ′ phase further increase. During long-term aging up to 440 h at 760 °C, the dispersed granular MC and M_6_C carbides dissolve and re-precipitate. By 2020 h of aging, flocculent carbides precipitate and non-continuous M_6_C and M_23_C_6_ accumulate at grain boundaries. After long-term aging at 815 °C for 440 h, flocculent carbides begin to precipitate within the grains. By 2020 h of aging, a large amount of flocculent carbides precipitate with significant coarsening and enrichment of the grain boundary carbides. Due to the insignificant coarsening of the γ′ phase as well as the enrichment and precipitation of the grain boundary carbides, the fatigue performance of the alloy decreases slightly after long-term aging.

## 1. Introduction

Powder metallurgy superalloys have rapidly emerged as the preferred materials for fabricating high-performance engine hot-end components, attributed to their uniform microstructure, absence of macroscopic segregation, fine grain size, and exceptional overall mechanical properties since inception [1,2]. As the thrust-to-weight ratio and turbine inlet gas temperature of aero engines continue to escalate, the demands for powder metallurgy superalloys utilized in vital hot-end components, such as turbine disks, have likewise intensified. In response, the United States and European nations have developed third-generation powder metallurgy superalloys, such as ME3, LSHR, and RR1000, etc., capable of enduring service temperatures ranging from 750 to 800 °C. These advanced alloys have been successfully integrated into turbine disks for aero engines, marking a significant milestone in the field [3,4,5]. Russia has successfully developed a novel series of powder metallurgy superalloys, designated as the BBII series [6]. China has developed the FGH series of powder metallurgy superalloys; yet, alloys suitable for 750–800 °C remain in the ongoing stages of development [2]. Our research team has developed a novel powder metallurgy superalloy, FGH4113A (also called WZ-A3), compared the typical powder superalloy LSHR, by elevating the Ti-to-Al ratio, thereby increasing the reverse stacking fault energy of γ′ to enhance the strength of the alloy, regulating solid solution elements of Mo and W, and adjusting Hf and B to ensure a balanced comprehensive performance at elevated service temperatures. This innovative alloy boasts mechanical performance levels that are comparable to those exhibited by internationally developed third-generation disk alloys [7].

The service environment of advanced aero engines is extremely harsh, which requires disk alloys to maintain high microstructural stability and mechanical properties during long-term service. The deterioration of the microstructure and mechanical properties during long-term high-temperature exposure have been a pivotal concern in the ongoing development of disk alloys. Gabb et al. [8] studied the microstructural and mechanical properties evolution of two third-generation powder metallurgy superalloys, LSHR and ME3, during long-term aging. Tian et al. [9] investigated the microstructural evolution of an FGH100L alloy. It was found that the tertiary γ′ particles within the supersolvus heat-treated samples underwent a consistent process of coarsening, while the primary carbide precipitates primarily transformed into M_23_C_6_ after prolonged aging. Notably, the carbides that precipitated along the grain boundaries exerted a pinning effect, thereby effectively enhancing the grain boundary strength. Cheng et al. [10] evaluated the coarsening behavior of the γ′ phase in Ni-based single-crystal superalloys during aging by an improved Ostwald ripening theory. Wang et al. [11] studied the γ′ precipitation behavior of an FGH97 alloy under long-term aging and summarized the evolution rules of three different sizes of γ′ phases. Our research team [12,13] previously compared an as-cast FGH4113 alloy and the typical third generation powder alloy RR1000 in a long-term aging study. It was found that FGH4113 exhibits superior creep characteristics at both 650 °C and 750 °C, along with enhanced microstructure stability after long-term aging. FGH113 has a lower C content than FGH113A. Certain studies have been carried out regarding the FGH4113A powder metallurgy superalloys with various states. The PPB precipitates were formed in a HIPed FGH4113A alloy by the diffusion of elements from the inter-dendritic regions to the powder particle surface, and they were determined to be a mixture of (Nb, Ti)C type carbides and (Hf, Zr)O_2_ type oxides [14]. The PPBs in the FGH4113A alloy can be efficiently eliminated via raising deformation temperature/true strain or reducing strain rate [15]. The effects of the extrusion rate on the evolution and mechanism of γ′ precipitation behavior, grain refinement, microtexture, and mechanical properties in the FGH4113A alloy have been studied [16]. Some studies have also been conducted on the microstructural stability of as-forged FGH4113A [17]. However, further investigation is needed to elucidate the impact of long-term aging on the microstructural evolution and fatigue characteristics of these alloys.

In this paper, FGH4113A was subjected to long-term aging treatment at 760 °C and 815 °C for up to 2020 h. The evolution of the alloy’s microstructure under different aging conditions and its effect on fatigue properties were studied. This study offers enhanced data support for the reliable long-term utilization of the FGH4113A alloy within the temperature range of 750–800 °C.

## 2. Materials and Methods

The experiments utilized a recently developed nickel-based powder metallurgy superalloy, FGH4113A. The nominal chemical composition (mass fraction, wt.%) is as follows: Co 19.0, Cr 13.0, Mo 4.0, W 4.0, Al 3.0, Ti 3.7, Nb 1.2, Ta 1.0, Hf 0.2, C 0.05, Zr 0.05, B 0.03, with the balance being Ni. The preparation process for the test disk involved vacuum induction melting to obtain a high-purity master alloy, argon gas atomization (AA) to produce powder, and sieving to achieve a powder size of ≤53 µm. The oxygen and the nitrogen contents of the powders were controlled below 100 ppm and 5 ppm, respectively. After inspecting for inclusions, the powder was loaded into a stainless steel can, degassed and sealed in vacuum by electron beam welding. The can was then processed by hot isostatic pressing (HIP) at 1150 °C and 150 MPa for 4 h. The HIP billets were hot-extruded (HEX) and isothermal-forged (IF), followed by solution and aging heat treatments to produce the final disk billet. The experimental specimens were cut from the disk billet. A supersolvus solution heat treatment was performed in an TITAN (H2) vacuum furnace (Ipsen, Souderton, PA, USA) at 1185 °C for 5 h, followed by argon gas cooling at a rate of approximately 150 °C/min. An aging treatment was then conducted at 815 °C for 8 h, followed by argon gas cooling at a rate of about 100 °C/min. The heat-treated specimens underwent long-term aging treatments in an SX-G36123 muffle furnace (HF-Kejing, Hefei, China) at 760 °C and 815 °C for 440 h and 2020 h, respectively.

After grinding, polishing, and etching, the grain size, γ′ phase, and carbide morphology of the specimens were observed using a Quantax eFlash HR Electron Back-Scatter Diffraction (EBSD) (Bruker, Berlin, Germany) and a Sigma 300 field emission Scanning Electron Microscope (SEM) (Carl Zeiss, Cambridge, UK). The specimens were etched by two kinds of solution of kalling’s and 3 mL HNO_3_ + 3 mL CH_3_COOH + 3 mL H_2_O + 1 mL HF, used to observe carbides and γ′ phase, respectively. An XFLASH6I60 Energy Dispersive Spectrometer (EDS) (Bruker, Berlin, Germany) was used for compositional analysis of the precipitates. Low-cycle fatigue tests were conducted according to GB/T 15248 [18], using an Instron8801 fatigue-testing machine (Instron, High Wycombe, UK). The fatigue test temperature was 760 °C, with stress-controlled mode, sinusoidal waveform, and a frequency of 0.33 Hz. The stress range was from −460 MPa to 880 MPa, and the test was terminated upon complete fracture of the specimen. The fracture surface morphology was examined using a SMZ1270 Optical Microscope (OM) (Nikon, Tokyo, Japan) and SEM. JMatPro version 7.0.0 (Ni database) was used to calculate equilibrium phase diagram, which was used to predict the precipitation and dissolution temperature of carbides.

## 3. Results

### 3.1. Microstructure Evolution of Alloy after Long-Term Aging

#### 3.1.1. Grain Structure and γ′ Phase

**Figure 1:**
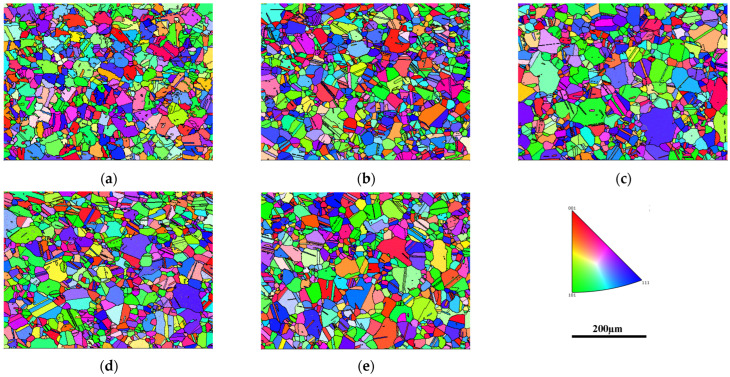
IPF maps images of FGH4113A after standard heat treatment and long-term aging, SHT (**a**); 760 °C/440 h (**b**); 760 °C/2020 h (**c**); 815 °C/440 h (**d**); 815 °C/2020 h (**e**).

The grain structures of the FGH4113A alloy after standard heat treatment (solution + aging, SHT) and long-term aging are shown in Figure 1. After SHT, the alloy exhibits a uniform grain distribution with an average grain size at approximately ASTM 7.2, and partial twins are visible within the grains, as shown in Figure 1a. After aging at 760 °C and 815 °C for 440 h and 2020 h, respectively, there are no significant changes observed in the grain morphology or size, maintaining a grain size around ASTM 7.5, as revealed in Figure 1b–e. The grain size statistics indicate that the grain size of the alloy fluctuates within 0.5 grade after SHT and long-term aging. After undergoing significant hot deformation processes, such as extrusion and forging, FGH113A experiences thorough dynamic recrystallization, resulting in grain refinement to ASTM 12–13. Subsequently, following the supersolvus solution heat treatment, the grains grow to ASTM 7–8. Long-term aging at 760 °C and 815 °C showed no obvious effect on the grain size. This conclusion is similar to that of Gabb et al. for LSHR [8].

**Figure 2:**
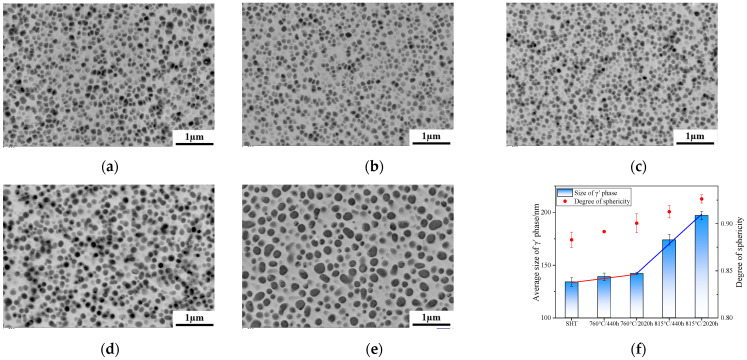
γ′ of FGH4113A after SHT (**a**); 760 °C/440 h (**b**); 760 °C/2020 h (**c**); 815 °C/440 h (**d**); 815 °C/2020 h (**e**); size and sphericity statistics chart (**f**).

The morphology, size, and sphericity statistics of the secondary γ′ strengthening phase within the grains under various conditions are presented in Figure 2. In the SHT state, the secondary γ′ precipitates within the grains are uniformly fine and primarily exhibit a rounded square morphology, with an equivalent diameter of 134 nm ± 4 nm. A small amount of finer tertiary γ′ can be observed within the matrix channels, as shown in Figure 2a. After aging for 440 h at 760 °C, the morphology of the secondary γ′ phase remains largely consistent with the SHT state, maintaining a uniform rounded square shape with a size of approximately 135 nm, as shown in Figure 2b. After aging for 2020 h at 760 °C, the size of the secondary γ′ does not increase significantly, but its sphericity increases, as shown in Figure 2c. After 440 h of aging at 815 °C, the morphology of the secondary γ′ phase transforms from a rounded square to a nearly spherical shape, resulting in an improvement in sphericity and a size of approximately 150 nm. When the aging time is extended to 2020 h, the size of the secondary γ′ phase increases further, as shown in Figure 2d,e. Figure 2f provides a clear visualization of the changing trends in the size and sphericity of the secondary γ′ phase under different aging conditions. The size of γ′ remains stable at 760 °C, as indicated by the red trend line, whereas at 815 °C, a notable increase in size is observed, as depicted by the blue trend line. As the temperature rises and the aging time elongates, the morphology of γ′ gradually evolves from a rounded square to a nearly spherical shape.

#### 3.1.2. Carbides

**Figure 3:**
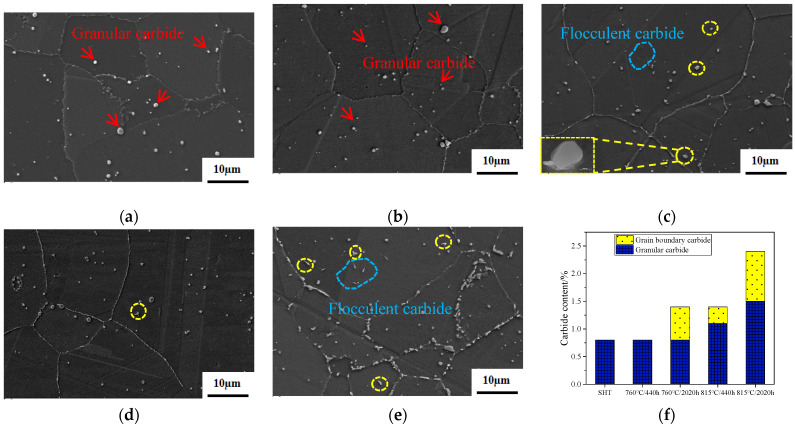
Carbide of FGH4113A3 after SHT (**a**); 760 °C/440 h (**b**); 760 °C/2020 h (**c**); 815 °C/440 h (**d**); 815 °C/2020 h (**e**); carbide distribution maps (**f**).

The type, morphology, distribution, and amount of carbides directly affect the mechanical properties of alloys. Figure 3 shows the morphological and statistical distribution of different types of carbides in the alloy, subject to varying aging conditions. After SHT, granular carbides are uniformly distributed within the grains, with no significant carbide enrichment observed at the grain boundaries, which exhibit a serrated pattern, as indicated by the red arrows in Figure 3a. After aging for 440 h at 760 °C, minor changes in the number and size of carbides are observed. Specifically, there is a trend of increasing carbide counts, accompanied by a reduction in the size of larger carbide particles, as indicated by the red arrow in Figure 3b. After 2020 h of aging at 760 °C, the size of the granular carbides dispersed within the grains decreases, as shown in Figure 3c. Notably, flocculent carbides start to emerge within the grains at this point. Some flocculent carbides precipitate randomly within the crystals, as indicated by the blue circles in Figure 3c, while others adhere to and grow on granular carbide particles, as highlighted by the yellow circles. Furthermore, there is a significant accumulation of carbides at the grain boundaries, resulting in a more straightened appearance of the grain boundaries.

After aging for 440 h at 815 °C, the changes in the number and size of granular carbides within the grains are similar to those observed at 760 °C for 440 h, as shown in Figure 3d. However, flocculent carbides attached to granular carbides begin to appear, as shown in the yellow circle in Figure 3d. After 2020 h of aging at 815 °C, there is a significant increase in the number of flocculent carbides within the grains, which could be categorized into two types: those growing dependent on granular carbides and those freely precipitated, as illustrated by the yellow and blue circles in Figure 3e, respectively. At this stage, the grain boundary carbides exhibit enrichment and coarsening, exhibiting a discontinuous distribution. The distribution patterns of carbides under varying aging conditions reveal that with a prolonged aging time and increased temperature, there is a significant increase in the content of carbides both within the grains and at the grain boundaries of the alloy, as evident in Figure 3f.

**Figure 4:**
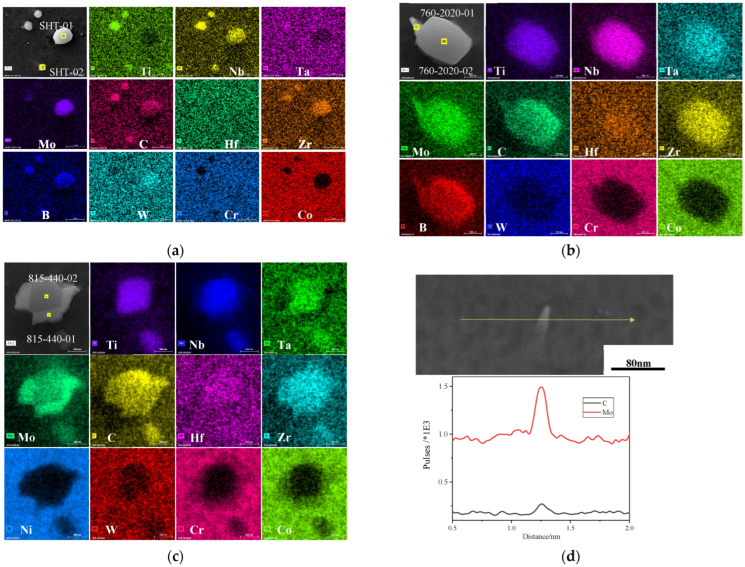
EDS maps for granular carbide of FGH4113A after SHT (**a**); 760 °C/2020 h (**b**); 815 °C/440 h (**c**); SEM micrograph and EDS line scan of flocculent carbide after 760 °C/2020 h (**d**). Different categories of carbides are highlighted with yellow boxes, and their respective compositions are enumerated in Table 1.

An EDS analysis of carbides precipitated within the grains is carried out, as shown in Figure 4. In the SHT state, the carbides are distributed in a dispersive granular form. According to Figure 4a, these granular carbides can be categorized into two types: MC carbides, primarily composed of strong carbon-forming elements such as Ti, Nb, Hf, and Ta; and M_6_C carbides, primarily composed of weak carbon-forming elements like W and Mo. Notably, both types of carbides exhibit a significant enrichment of the B element. Figure 4b,c present the precipitated phases associated with attached granular carbides aging at 760 °C for 2020 h and 815 °C for 440 h, respectively. The attached granular carbides are enriched in elements such as Ti, Nb, Hf, and Ta, whereas the attached precipitated carbides primarily concentrate W and Mo. A comparison of the energy spectrum composition reveals a significant difference in the content of Ti, Nb, Hf, Ta, W, and Mo between the two types of carbides. Figure 4d illustrates a line scan of freely precipitated carbides within the alloy, revealing a concentration of Mo and C. Table 1 is the composition table of granular carbides in Figure 4a–c, the specific location is marked with a yellow box, which shows that carbides are different significantly in terms of Ti, Nb, Hf, Ta, W, and Mo content.

**Figure 5:**
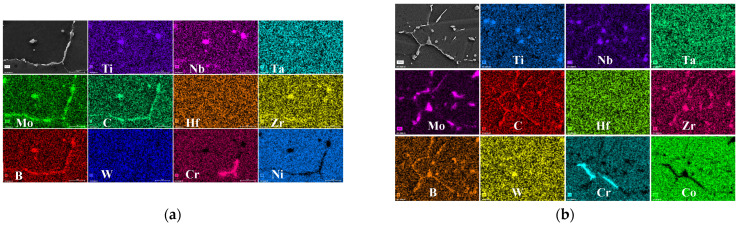
EDS maps for grain boundary carbide of FGH4113A after 760 °C/2020 h (**a**); 815 °C/2020 h (**b**).

As depicted in Figure 5, there exists a significant difference in the morphology of grain boundary carbides between the conditions of aging at 760 °C for 2020 h and 815 °C for 2020 h. After aging at 760 °C for 2020 h, high-Cr dendritic carbides were observed to precipitate at the grain boundaries of the alloy. Similarly, high-Cr dendritic carbides exhibiting comparable morphologies and compositions were also noted under the conditions of aging at 815 °C for 2020 h. After prolonged aging at both 760 °C and 815 °C, the grain boundary carbides commenced a transformation process, evolving from MC carbides to M_23_C_6_ carbides.

### 3.2. Low-Cycle Fatigue Properties

**Figure 6:**
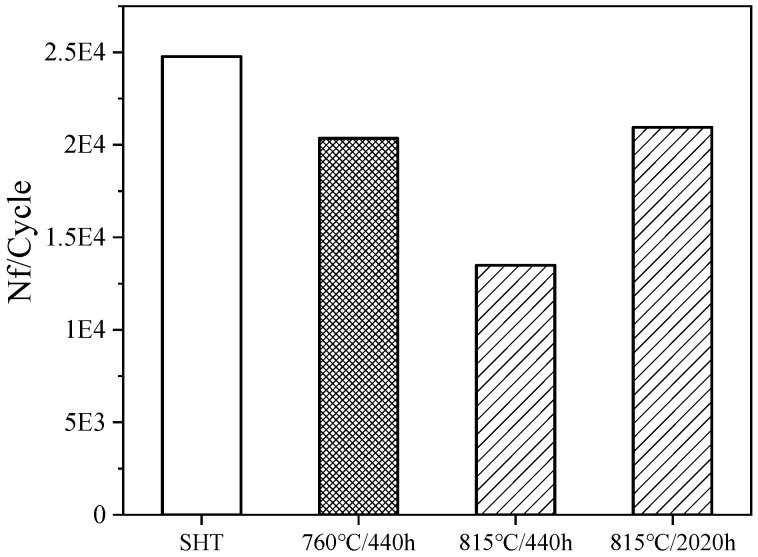
Low-cycle fatigue life of FGH4113A at 760 °C.

The low-cycle fatigue (LCF) property at working temperatures is a crucial characteristic of disk alloys. The LCF life of the FGH4113A alloy at 760 °C was evaluated after SHT, and after long-term aging at 760 °C for 440 h, 815 °C for 440 h, and 815 °C for 2020 h, with the results presented in Figure 6. In the SHT state, the alloy achieved a fatigue life of 24,770 cycles. After long-term aging at 760 °C for 440 h, a slight reduction in the LCF life was observed, averaging 20,351 cycles. However, when aged at 815 °C for 440 h, the LCF life decreased significantly to 13,489 cycles, which was due to non-metallic inclusion unfortunately. Notably, extending the aging time to 2020 h at 815 °C resulted in a substantial improvement in fatigue life, comparable to that observed after aging at 760 °C for 440 h. Analysis of the reduction in low cycle fatigue life across different aging conditions revealed decreases of 18%, 46%, and 15% after aging at 760 °C for 440 h, 815 °C for 440 h, and 815 °C for 2020 h, respectively, compared to the SHT state. Gabb et al. [8] compared the LCF life of the third-generation powder alloy LSHR at 760 °C before and after undergoing long-term aging, to assess microstructure aging effects. Compared to the specimens that were not thermally exposed, the fatigue lives decreased by approximately 25% after aging at 815 °C for 2020 h. This reduction can be attributed to the decrease in strength of the alloy following long-term aging.

Upon microscopic examination of the fracture surfaces of the FGH113A alloy LCF specimens, it was discovered that only one out of the eight fatigue fractures, specifically the one aged at 815 °C for 440 h, exhibited an inclusion-type fatigue origin. This non-metallic inclusion measured 95 µm × 55 µm. The remaining seven fractures, on the other hand, exhibited platform-type crack origins, all originating from the surface of the specimens.

**Figure 7:**
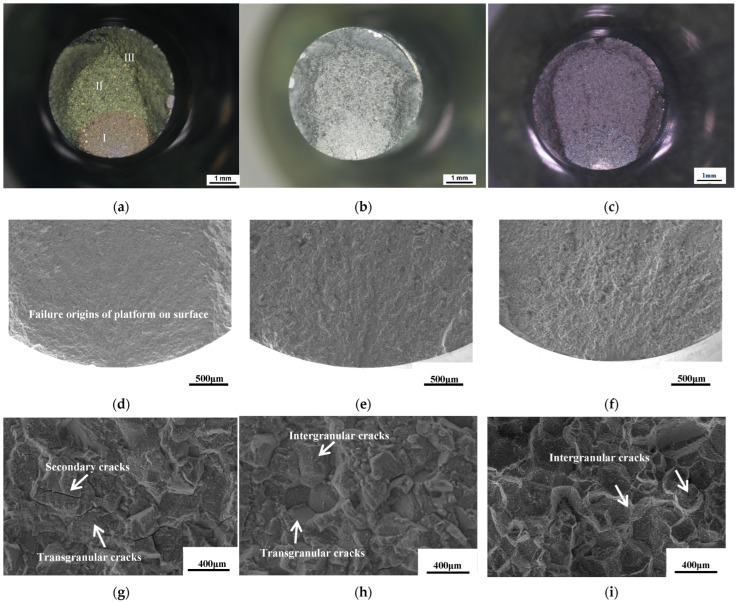
Low-cycle fatigue fracture morphology of FGH4113A (**a**,**d**,**g**) SHT; (**b**,**e**,**h**) 760 °C/440 h; (**c**,**f**,**i**) 815 °C/2020 h.

The macroscopic morphology of the fracture of the LCF specimens in the SHT state, aging at 760 °C for 440 h, and 815 °C for 2020 h conditions are shown in Figure 7a–c. As depicted in Figure 7a of I, II, and III, all three conditions exhibit distinct zones pertaining to crack initiation, propagation, and ultimate rupture. Notably, the cracks emanate from the specimen’s surface, devoid of any metallurgical defects that could have served as fatigue sources. Instead, they exhibit characteristic features of typical platform fatigue sources, as clearly illustrated in Figure 7d–f.

The cracks observed on the surface platform arise from the gradual accumulation of plastic damage within the slip bands. FGH4113A, characterized as a nickel-based alloy with a high degree of alloying and a low stacking fault energy, is prone to planar slipping under cyclic loading conditions. This slipping behavior gives rise to the formation of slip bands. Subsequently, under alternating stress, these slip bands produce “intrusions” and “extrusions” on the surface of the specimen. After a certain number of cycles, localized persistent slip bands form, promoting crack nucleation on the surface, resulting in platform fatigue sources [19].

Significant disparities exist in the crack propagation zones among the three conditions. Specifically, the SHT state demonstrates characteristic transgranular attributes, including prominent fatigue striations and secondary cracks, as depicted in Figure 7g. The aging states at 760 °C for 440 h and 815 °C for 2020 h exhibit a blend of intergranular and transgranular fractures. Notably, the latter aging condition exhibits a greater abundance of dimples, as evident in Figure 7h,i. This indicates that the alloy aging at 815 °C for 2020 h has higher plasticity.

## 4. Discussion

### 4.1. The Evolution of the γ′ Phase

**Figure 8:**
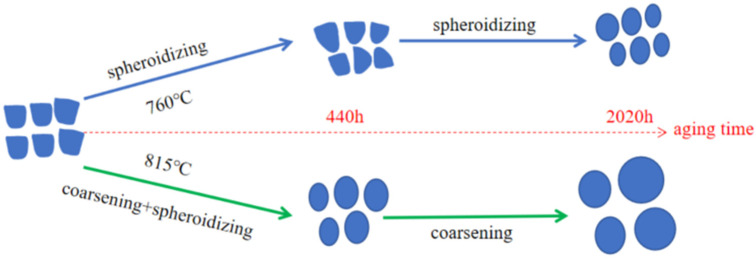
Schematic diagram for γ′ evolution at different aging conditions.

The evolution of the γ′ phase under varying aging conditions is shown in Figure 8. Specifically, as the aging duration elongates at 760 °C, the γ′ phase undergoes morphological alterations, evolving from rounded squares in the SHT state to rounded triangles, and ultimately approximating spherical shapes. The size of the γ′ phase remains consistent, approximately maintaining a dimension of 130 nm throughout this process. As the aging at 815 °C, both the spheroidization and coarsening of the γ′ phase occur concurrently, with the duration of aging leading to these transformations. Once spheroidization stabilizes, the size continues to increase as the aging time extends, and the coarsening process persists.

The γ′ solvus temperature of FGH4113A is approximately 1158 °C. Upon undergoing supersolvus heat treatment at 1185 °C, followed by rapid cooling, the coarse primary γ′ phase dissolves entirely, while the fine secondary γ′ phase rapidly precipitates and distributes uniformly within a short time. The dissolution of γ′ is primarily driven by the diffusion of solute atoms, which is further influenced by factors such as the heating temperature, holding time, and the initial state of the alloy [20]. After long-term aging at 760 °C for 2020 h, the secondary γ′ phase exhibits a comparable size to its SHT state counterpart, albeit with a slight spheroidization trend. This observation suggests that Ostwald ripening has not taken place. At the operational temperature of 760 °C, the diffusion rate of the γ′-forming elements, including Al and Ti, remains exceptionally low, thereby preserving the exceptional stability of the strengthening phase.

In contrast, during long-term aging at 815 °C for 440 h, the secondary γ′ phase begins to coarsen rapidly. As the aging time extends to 2020 h, the secondary γ′ phase undergoes a significant increase in size, accompanied by a decrease in its volume fraction, indicating the occurrence of Ostwald ripening at this temperature. Smaller γ′ phases gradually dissolve into the matrix, while solute atoms within the matrix diffuse into larger γ′ phases, leading to their growth and a reduction in the overall free energy. Consequently, FGH4113A becomes more susceptible to Ostwald ripening as the aging temperature rises from 760 °C to 815 °C.

In nickel-based superalloys, the γ and γ′ have almost identical elastic constants, allowing the contribution of chemical-free energy to be neglected. In alloy systems with a large-volume fraction of precipitates, the driving force for coarsening is as follows [21]:

(1) $$∆E_{total}=∆E_{str}+{∆E}_{surf}+{∆E}_{int}$$

where $E_{total}$ is the total system energy, $E_{str}$ is the elastic strain energy due to lattice mismatch between the γ′ precipitates and the γ matrix, $E_{surf}$ is the interfacial energy of the γ′ precipitates, expressed as 2τ/r (where τ is the interfacial tension and r is the precipitate radius), and $E_{int}$ is the elastic interaction energy between the γ′ precipitates.

The elastic strain energy $∆E_{str}$ and elastic interaction energy $∆E_{int}$ can be calculated as follows [22,23]:

(2) $$∆E_{str}+∆E_{int}=\alpha VE_{1}\;=\alpha V\left(-\frac{1}{2}{\left(C_{11}+C_{12}\right)}^{2}C_{11}-C_{12}-{2C}_{44}\frac{\in_{unconstrained}^{2}}{C_{11}({2C}_{11}+C_{12}+{2C}_{44})}\right)$$

where V is the volume fraction of the γ′ precipitates; α is related to the shape (0.709 for spherical, 0.436 for cubic); $E_{1}$ is the elastic energy density; $C_{11}$, $C_{12}$, and $C_{44}$ are the cubic elastic constants of the γ′ phase. Equation (2) shows that the total elastic energy is linearly related to the square of the unconstrained lattice strain $\in_{unconstrained}^{2}$.

The morphology of the precipitates is primarily determined by the minimization of the sum of the elastic energy $E_{str}$ and the interfacial energy $E_{surf}$. During long-term aging, γ′ precipitates adopt an optimal morphology to achieve a low energy state and maintain structural stability [24]. The preferred morphology is the stable shape when the sum of elastic energy and interface energy is minimized. For FGH4113A, the transition of the near-cubic γ′ phase into a near spherical shape results in a decrease in both interface energy and elastic strain energy. The near spherical configuration of the γ′ phase is a result of transitioning from an initially near cubic metastable state to a stable state.

During the long-term aging of FGH4113A at 760 °C for 2020 h, the system’s interfacial energy decreases, while the elastic strain energy does not increase, preventing γ′ from splitting and coarsening, only resulting in noticeable spheroidization.

Chen et al. [22] studied the coarsening behavior of γ′ in RR1000 during short-term aging at 800 °C and concluded that the elastic energy resulting from local compositional changes directly affects the precipitation morphology of γ′. The complex inter-diffusion between the matrix and the precipitates determines the coarsening rate of the precipitate-strengthening phase. In terms of energy, splitting is favorable when the reduction in elastic strain energy exceeds the increase in interface energy. Elastic energy and interface energy have been used to explain the splitting of cubic γ′ in nickel-based superalloys [25]. However, the splitting process occurs only in the early stages of aging. In the later stages of aging, particle aggregation reduces lattice strain within the matrix, similar to the evolution of γ′ in the alloy, as shown in Figure 3 [26].

At the 815 °C aging temperature, the spheroidization process of the γ′ phase is similar to that at 760 °C, but under high-temperature induction, the atomic diffusion is intense, which makes the size of γ′ slightly increase, so γ′ spheroidization and coarsening occur at the same time. During the long-term aging of LSHR at 815 °C for 2020 h, the evolution of the γ′ phase is similar to that of FGH4113A, while precipitate size actually decreased for ME3. This was apparently related to the stability of the highly extended, lobed precipitates in ME3 [8].

### 4.2. The Evolution of Carbides

**Figure 9:**
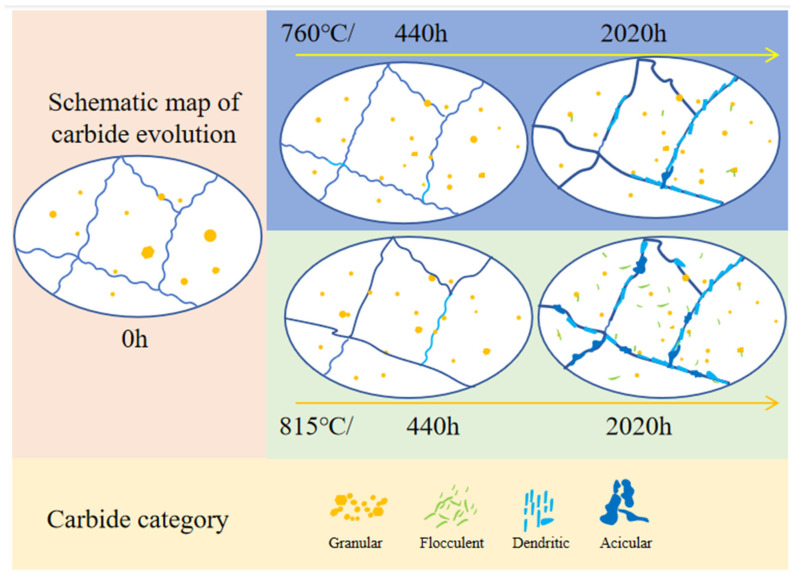
Schematic diagram for carbide evolution at different aging conditions.

The evolution of carbides during long-term aging is shown in Figure 9. The alloy contains dispersed MC and M_6_C granular carbides, with M_6_C carbides typically being larger, reaching up to 1 µm in the SHT state. The grain boundaries exhibit a distinct serrated pattern. After long-term aging at 760 °C for 440 h, the granular carbides remain dispersed throughout the alloy. The size of the granular carbides decreases, but their number increases, in comparison to their SHT state. Some large M_6_C carbides slowly dissolve into the matrix, while new granular carbides precipitate under this condition. Upon reaching an aging time of 2020 h, flocculent carbides commence precipitation, exhibiting a random and dispersed distribution throughout the material. These carbides are primarily enriched with Mo and are classified as belonging to the M_6_C. A significant amount of carbides precipitate at the grain boundaries primarily in two forms: one is more prevalent, dendritic carbide, which is enriched with strong carbide-forming elements and classified as MC; and the other one is less common, acicular carbide, enriched with Cr and classified as M_23_C_6_. Notably, the grain boundaries exhibit a tendency to straighten.

Upon prolonged aging at 815 °C, the dissolution and precipitation rates of carbides undergo a significant acceleration. At the 440 h, the flocculent carbides become visible, accompanied by a notable straightening of the grain boundaries. As the aging duration extends to 2020 h, the flocculent carbides become widely dispersed throughout the alloy. Dendritic and acicular carbides, often larger in size, are increasingly observed along the grain boundaries. The transformation of carbides is a diffusive phase transition in the superalloys. The atoms entered the new phase quickly by thermal activation to overcome the energy barrier, nucleated, and grew up during long-term aging at 815 °C.

**Figure 10:**
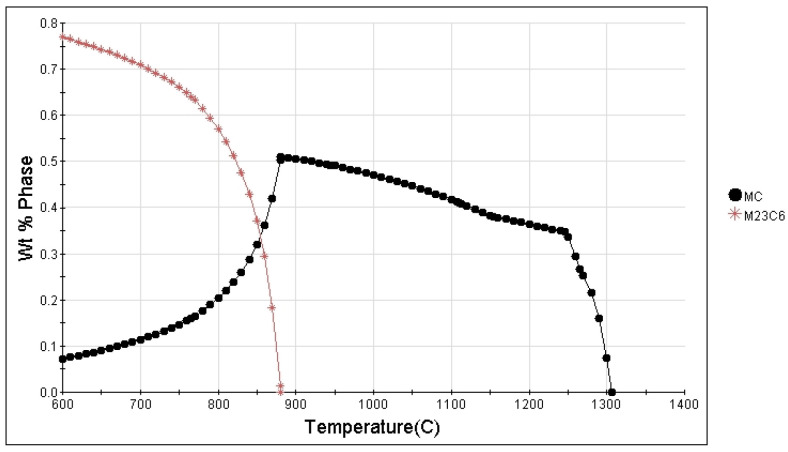
Carbide precipitation diagram calculated by JMatPro.

The carbide precipitation behavior of the FGH4113A alloy, calculated using JMatPro software version 7.0.0 (Ni database) based on thermodynamic equilibrium, is shown in Figure 10. MC carbides, primarily consisting of Hf, Ta, Nb, Zr, and Ti, precipitate at 1308 °C. These carbides commence decomposition into M_23_C_6_ carbides, which are predominantly composed of Cr and Mo at 882 °C. However, due to the rapid solidification process inherent in atomization powder production, the actual solidification does not yield a structure with full equilibrium. Additionally, the primary carbides in FGH4113A alloy undergo transformation throughout powder production, hot deformation, and supersolvus heat treatment. Therefore, MC and M_6_C carbides are observed, but not M_23_C_6_ in the SHT state.

The temperature range in which M_6_C exists is 760–1150 °C in superalloys, typically peaking in precipitation at 870–1100 °C. M_23_C_6_ carbides exist in the range of 650–1100 °C, with a precipitation peak at 850–980 °C, depending closely on the alloy composition and precipitation conditions. The relationship between relative *Cr* content $R_{Cr}$ and the type of carbide precipitation has been studied, and the following three empirical formulas have been proposed [27]:

(3) $$R_{Cr}=\frac{Cr\;at.\%}{(Cr+Mo+0.7\;W)\;at.\%}\;\mathrm{When}\;R_{Cr}<0.72,\;M_{6}C\;\mathrm{forms}.$$

(4) $$\mathrm{When}\;R_{Cr}>0.82,\;M_{23}C_{6}\;\mathrm{forms}.$$

(5) $$R_{Cr}<0.82,\;the\;type\;of\;carbide\;formed\;varies\;with\;heat\;treatment.$$

Guo [28] validated these three empirical formulas with 12 types of superalloys. $R_{Cr}$ equals to 0.8 for FGH4113A, hence the type of carbide formed depends on heat treatment. MC and M_6_C carbides were indeed generated in this study. During long-term aging, large particles of metastable M_6_C gradually dissolve into the matrix, which results in increasing the W and Mo content in the matrix. When the W and Mo contents reached supersaturation, the high Mo flocculent M_6_C precipitated from the alloy under high temperature induction, and some also attached to the MC grain carbides. This is due to the reduced stability of the MC as W and Mo enter it, allowing it to produce a degradation reaction; the reaction formula can be written as follows: MC + γ → M_6_C + γ′ [29].

Grain boundaries, as a type of crystal defect, become important nucleation points for carbides during long-term aging and coarsening. After dissolving into the matrix, the elements are redistributed and diffuse into M_6_C carbides at the grain boundaries, leading to the coarsening of the M_6_C carbides [30]. M_6_C carbides are usually rich in W and Mo and poor in Cr [31]. However, in high-Cr superalloys, Cr can also enter M_6_C carbides, and when the Cr concentration in M_6_C is sufficiently high, decomposition may occur under certain driving forces [30,32,33]. M_6_C is in a metastable state, with the possibility of reaction of: M_6_C→M_23_C_6_ + Me(W, Ni, Cr, Mo) [32]. Additionally, some M_23_C_6_ can precipitate from a supersaturated γ solid solution in the form of γ→M_23_C_6_ + γ′. M_23_C_6_ generally nucleates at grain boundaries to reduce the total free energy of the solid solution. During nucleation and growth, it absorbs a large amount of Cr, W, Mo, and other elements from the solid solution, enriching the surrounding environment with Al, Ti, and Ni elements that form γ′ [34].

From thermodynamic and kinetic analyses, an addition of 0.04 wt.% C in FGH4113A results in the precipitation of M_6_C carbides during long-term aging. These carbides precipitate both at grain boundaries and within grains. However, because dendritic M_6_C carbides at grain boundaries coarsen and consume a large amount of W and Mo, there are fewer flocculent carbides near the grain boundaries. This conclusion is similar to that of Huang et al. [30].

### 4.3. Effect of Long Term Aging on the Fatigue Properties

The LCF fracture characteristics of powder metallurgy superalloys, particularly the morphology of crack origins, are closely related to the fatigue life of the alloy [35]. In the previous analysis of eight fatigue fracture specimens of FGH4113A, only one specimen aged at 815 °C for 440 h exhibited an inclusion-type fatigue source, while the remaining seven exhibited platform-type crack sources. When the size of the inclusion attains a critical threshold, it significantly impacts the fatigue life, with the LCF life decreasing linearly as the inclusion size increases beyond this threshold [36]. Notably, the fatigue life of specimens aged at 815 °C for 440 h is relatively lower compared to other conditions, with the presence of inclusions being a significant factor contributing to this reduction.

In addition to the inclusion, the factors that affect the fatigue life of powder superalloys generally include alloy composition, grain size, γ′ phase, and carbides [37,38]. In this study, the alloy composition of all fatigue specimens was consistent, so only changes in the alloy’s microscopic structure, including grain size, γ′ phase, and carbides, were considered. Comparing the microstructures of FGH4113A in the SHT state and after long-term aging at 760 °C for 440 h, 815 °C for 440 h, and 815 °C for 2020 h, it can be seen from Figure 1 that the grain size of the alloy in the four states has no significant change, remaining between 20 and 23 µm; the grain size fluctuates no more than ASTM 0.5, hence the grain size has little effect on fatigue life.

After aging at 760 °C for 440 h, the secondary γ′ phase precipitated within the grain of the alloy remained square in shape and had essentially no difference in size compared to the standard heat-treated state, as shown in Figure 2. However, long-term aging at 815 °C after 440 h and 2020 h, the secondary γ′ phase began to spheroidize with the size increasing from 134 nm ± 4 nm in the SHT state to 174 nm ± 5 nm and 197 nm ± 4 nm, respectively. The change in γ′ is more pronounced under long-term aging at 815 °C compared to that at 760 °C. It is observed in Figure 7h that after long-term aging at 815 °C for 2020 h, there were more dimples in the fracture surface of LCF, due to the coarsening of γ′ and the recovery of the matrix structure, making the alloy have higher plasticity.

Figure 3 reveals that the dispersed granular carbides show no significant changes at 760 °C for 440 h and 815 °C for 440 h, compared to the SHT state. After aging at 815 °C for 2020 h, the number of dispersed granular carbides decreases, while the grain boundary M_6_C carbides increase and coarsen. At the same time, M_23_C_6_ with high Cr also appears in the grain boundary [39,40].

**Figure 11:**
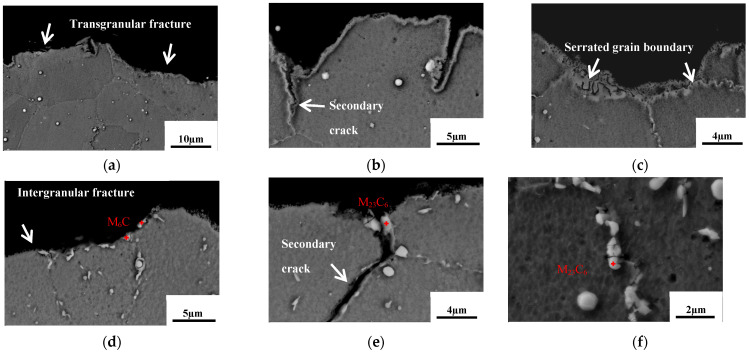
Longitudinal sectional morphology of the fatigue fracture in different states: SHT (**a**); 760 °C/440 h (**b**); 815 °C/440 h (**c**); 815 °C/2020 h (**d**–**f**). The locations of grain boundary carbides in the Figure (**d**–**f**) are indicated by a red cross, along with their respective categories.

The longitudinal sectional morphology of the fatigue fracture surfaces in different states is shown in Figure 11. In the SHT state, the fatigue fracture mainly exhibits transgranular fracture, as shown in Figure 11a. After aging at 815 °C for 2020 h, the fracture primarily propagates along the grain boundaries, demonstrating significant intergranular fracture, as shown in Figure 11d. Secondary cracks propagating along the serrated grain boundaries at 760 °C for 440 h are shown in Figure 11b. After aging at 815 °C for 440 h, a fracture is observed in proximity to the serrated grain boundary, which exhibited robust bonding characteristics, and thus did not propagate along this grain boundary at high temperatures and stresses, as shown in Figure 11c. Figure 11e displays secondary cracks propagating along carbide-enriched grain boundaries, with M_6_C carbides along the fracture propagation direction. Near the fracture surface, carbides break along the axial stress direction, as shown in Figure 11f. M_6_C carbides are brittle and prone to fracture under repeated axial stress [41]. The interface between M_6_C carbides and the matrix has weak cohesive strength, leading to interface fracture under axial forces which becomes a path for crack propagation.

Typically, M_6_Cs serve as crack initiation sites and crack propagation paths. As shown in Figure 6, the LCF life of the alloy under different aging conditions indicates that the fatigue life of the alloy after long-term aging decreased compared with the SHT state. Under conditions where the crack origins are all platform, the serrated grain boundaries contribute to the alloy’s strength. However, carbide enrichment at the grain boundaries accelerates crack propagation to some extent, reducing the LCF life of the alloy.

Studies have shown [42] that among grain size, γ′ phase, and carbides, grain size has the greatest effect on the fatigue crack growth rate. Decreasing grain size and increasing γ′ phase and carbide size will decrease the resistance of the alloy to fatigue cracks. Consequently, the alloy exhibits the highest fatigue life in the SHT state. After long-term aging at 760 °C for 440 h and 815 °C for 2020 h, the reduction in the LCF life is within 20%, primarily due to the growth of the γ′ phase and carbide enrichment at the grain boundaries. After long-term aging at 815 °C for 2020 h, the γ′ phase coarsens and the matrix structure recovers, which improves the plasticity of the alloy. This can be used to understand that although the γ′ phase coarsens and the carbides at grain boundaries are enriched after aging at 815 °C for 2020 h, the LCF life of the alloy is equivalent compared to that of the alloy aged at 760 °C for 440 h. The presence of non-metallic inclusions is the main reason for the significant reduction in the fatigue life at 815 °C for 440 h. Therefore, it can be considered that long-term aging has a relatively minor impact on the LCF performance of FGH4113A alloy.

## 5. Conclusions

A new nickel-based powder metallurgy superalloy, FGH4113A, was subjected to long-term aging at 760 °C and 815 °C for up to 2020 h to observe its microstructure evolution and perform fatigue performance tests. This study provides more data support for the long-term application of the FGH4113A alloy at a temperature range of 750 °C~800 °C. The following conclusions can be drawn:

(1) The long-term aging at 760 °C or 815 °C up to 2020 h revealed no effect on the grain size of the alloy, which maintained unchanged at about ASTM 7.5.
(2) After the long-term aging at 760 °C for 2020 h, the size of the secondary γ′ phase showed no significant change, holding at about 130 nm. But the morphology of the γ′ phase changed from the rounded squares to nearly spherical shapes. After aging at 815 °C for 440 h, the secondary γ′ phase coarsening and spheroidizing took place. The size of the γ′ phase increased to about 200 nm after 2020 h, and the morphology also changed to a near-spherical shape.
(3) After long-term aging at 760 °C for 2020 h, dispersed MC carbides transformed into high-Mo flocculent M_6_C carbides and noncontinuous M_6_C. In the same time, M_23_C_6_ carbides began to enrich at the grain boundaries. After aging at 815 °C for 440 h, flocculent carbides started precipitating within the grains. The amount of flocculent carbides within the grains kept on growing, and the grain boundary carbides became coarser after 2020 h.
(4) Compared to the SHT heat treatment state, the 760 °C LCF life of the alloy decreased after long-term aging. The reductions in fatigue life after aging 760 °C for 440 h and 815 °C for 2020 h showed no significant difference. The maximum reduction in LCF life is less than 20% for the conditions in this study. The little reduction in fatigue life after long-term aging is associated with the insignificant coarsening of the γ′ phase and the enrichment of carbides at the grain boundaries.

## Figures and Tables

**Table 1:** Composition of granular carbide of FGH4113A by EDS (wt.%).

Alloy Status	Al	Ti	Cr	Co	Ni	Zr	Nb	Mo	Hf	Ta	W	Ti, Nb, Hf, Ta	W, Mo
SHT-01	1.4	3.4	16.2	9.3	21.9	1.6	2.8	24.1	0.2	0.5	18.6	6.9	42.7
SHT-02	2.8	8.5	11.0	16.1	40.7	0.5	6.6	3.8	0.5	6.0	3.4	21.7	7.2
760-2020-01	2.7	5.6	12.6	18.7	39.3	0.4	3.3	8.4	0.3	2.0	6.8	11.2	15.1
760-2020-02	1.5	15.9	7.8	10.7	25.8	1.1	13.3	2.9	2.5	15.4	3.1	47.1	6.0
815-440-01	2.0	5.5	12.3	17.9	31.6	0.5	4.0	11.9	0.7	3.1	10.6	13.3	22.5
815-440-02	1.5	14.5	8.1	11.3	26.8	0.9	12.3	5.5	1.7	13.5	3.9	42.0	9.4

## Data Availability

The original contributions presented in the study are included in the article, further inquiries can be directed to the corresponding authors.
